# Interlayer Electrons Polarization of Asymmetric Metal Nanoclusters/g‐C_3_N_4_ for Enhanced Microwave Therapy of Pneumonia

**DOI:** 10.1002/advs.202301817

**Published:** 2023-05-10

**Authors:** Yuan Li, Shuilin Wu, Yufeng Zheng, Zhaoyang Li, Zhenduo Cui, Hui Jiang, Shengli Zhu, Xiangmei Liu

**Affiliations:** ^1^ School of Materials Science & Engineering Peking University Yiheyuan Road 5# Beijing 100871 China; ^2^ School of Materials Science & Engineering The Key Laboratory of Advanced Ceramics and Machining Technology by the Ministry of Education of China Tianjin University Tianjin 300072 China; ^3^ Biomedical Materials Engineering Research Center Hubei Key Laboratory of Polymer Materials Ministry‐of‐Education Key Laboratory for the Green Preparation and Application of Functional Materials School of Materials Science and Engineering Hubei University Wuhan 430062 China; ^4^ School of Health Science & Biomedical Engineering Hebei University of Technology Xiping Avenue 5340 Beichen District Tianjin 300401 China

**Keywords:** g‐C_3_N_4_, interlayer electron, microwave disinfection, nanoclusters doping, pneumonia therapy

## Abstract

Interlayer interactions in two dimensional (2D) materials promote catalytic performance but often depend on the transport of inter rather than intralayer electrons. In this study, it is found that asymmetric metal‐nanocluster‐doped 2D g‐C_3_N_4_ greatly enhances catalytic performance by inducing microwave excitation of interlayer electron delocalization, resulting in a polarization of interlaminar charge transport for microwave disinfection and pneumonia therapy. Asymmetric Fe and Cu nanocluster doping (DCN‐FeCu) enables g‐C_3_N_4_ to generate interlayer electrons under microwave irradiation, leading to interlayer polarization processes and electron delocalization effects, thus enhancing the interlayer migration efficiency of electrons. It also improves impurity energy levels and leads to a decrease in work function, allowing DCN‐FeCu to produce microwave carriers across the photoelectric potential barrier under low‐energy microwave radiation (2.45 GHz). This asymmetric doping modulation produces layer number‐dependent microwave electron excitations that are verified using multi‐metal doping. Therefore, structurally modulated asymmetric doping of 2D materials with interfacial spatial effects can provide efficient microwave disinfection and pneumonia therapy.

## Graphical abstract

Asymmetric metal nanoclusters‐doped 2D g‐C_3_N_4_ is designed, which greatly enhances the microwave antibacterial performance by interlayer electron polarization and microwave‐modulated energy band changes leading to 3D charge transport. This asymmetric metal nanocluster doping enables g‐C_3_N_4_ to generate interlayer carriers under the microwave, leading to interlayer electron polarization and electron delocalization effects, thus enhancing the interlayer migration efficiency of electrons.

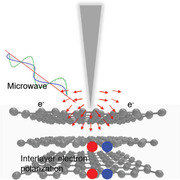

## 1 Introduction

Two dimensional (2D) materials, as emerging nanomaterials in the 21st century, have extensive applications for energy storage devices, chemical sensors, optoelectronic devices, and condensed matter physics due to their outstanding optoelectronic properties.^[^ ^1^, ^2^, ^3^, ^4^, ^5^, ^6^, ^7^ ^]^ 2D materials have planar dimensions and atomic‐level thicknesses that provide a large specific surface area—a property that has prompted extensive research for their use in devices such as catalyzers and capacitors.^[^ ^8^, ^9^, ^10^, ^11^ ^]^ In recent years, graphitic phase carbon nitride (g‐C_3_N_4_), which has a graphene‐like layered structure, has received considerable attention^[^ ^12^, ^13^, ^14^, ^15^ ^]^ as a typical n‐type semiconductor with good conduction band electron reduction ability and enormous potential for catalytic hydrogen production.^[^ ^16^, ^17^ ^]^ However, CN has a large band gap (about 2.7 eV) and weak absorption in the visible light waveband.^[^ ^18^ ^]^ Moreover, its low separation efficiency of photogenerated electron–hole pairs and slow carrier transport result in low catalytic activity.^[^ ^19^, ^20^ ^]^ To solve this problem, CN must be modified to improve its electron transport activity.

Metal doping is achieved by introducing ions into the inner lattice of semiconductor catalysts.^[^ ^21^, ^22^ ^]^ The band gap narrows due to the ability of the doped metal element to produce a sender energy level above the valence band or a host energy level below the conduction band of the semiconductor.^[^ ^23^ ^]^ In addition, doping can create electron traps on the surface of the substrate to trap electrons that emerge from inside the material, inhibit carrier complexation, and enhance catalytic efficiency.^[^ ^24^, ^25^ ^]^ When the metal and CN form a heterogeneous interface, the inconsistency of the Fermi energy level drives the CN electrons to the doped metal surface until the Fermi energy levels of the two reach equilibrium.^[^ ^17^, ^26^ ^]^ The electrons then accumulate on the metal surface, reducing the CN electrons and effectively inhibiting the complexation of CN photogenerated carriers.^[^ ^27^, ^28^ ^]^ However, the doping methods developed to date all employ dissymmetric doping, which does not maximize the catalytic properties or improve the connection between the doping structure of metal elements and the catalytic enhancement mechanism. In addition, carbon‐related nanosheets or nanospheres are receiving increasing attention due to their better dielectric effects, and the construction of interfacial effects or modulation of the structure can greatly improve their microwave absorption performance.

Microwaves, as an auxiliary external field, can have thermal or non‐thermal effects on diverse reactions such as biomass synthesis or semiconductor photocatalytic reactions.^[^ ^29^, ^30^, ^31^ ^]^ Thermal effects mainly increase the rate of reactions by increasing the activation energy of the reacting molecules, but the role of non‐thermal effects in catalytic reactions is unclear.^[^ ^32^, ^33^ ^]^ Since microwave energy is low compared to that of light, it is difficult to break the incident frequency threshold of the conventional photoelectric barrier when a semiconductor absorbs microwave irradiation, making it difficult to achieve microwave absorption below the height of the barrier.^[^ ^34^, ^35^ ^]^ CN as a carbon‐based material has a certain dielectric effect and good wave absorption.^[^ ^36^ ^]^ However, its catalytic properties and catalytic mechanism for producing nonthermal effects under microwave irradiation remain unknown.

Previous works have shown that copper cysteamine and the TiO_2_ nanoparticles TPEPy‐I and TPEPy‐PF6 can act as microwave absorbers.^[^ ^36^ ^]^ They are able to produce free radicals, such as singlet oxygen species, under microwave irradiation, thus killing tumor cells. Inorganic materials and inorganic–organic hybrids have certain microwave absorption capacities. In this study, we found that tuning conventional doping to modulate the symmetry of the overall structure of g‐C_3_N_4_ nanosheets enhanced their charge transport properties under microwave irradiation, mainly based on the enhanced transport of inter rather than intralayer electrons. Moreover, we found that the doped asymmetric g‐C_3_N_4_ nanosheets underwent charge transport under microwave irradiation due to the reduced work function, which led to the appearance of impurity energy levels and easier microwave excitation phenomena. We took advantage of CN's interlayer electron polarization, which can interact with an alternating microwave field, to achieve delocalized electron migration and efficient microwave catalysis by introducing dissymmetric metal Fe and Cu nanoclusters of doped modified CN (DCN‐FeCu). We achieved this asymmetric doping by controlling the presence of only one type of nanocluster (Fe or Cu) on a layer of CN, thus generating inter and intralayer inconsistencies in CN and leading to differences in polarization coupling between layers. This doping of Fe and Cu nanoclusters resulted in the polarization of interlayer electrons in CN due to the electron delocalization effect, thus enhancing the interlayer carrier migration under microwave irradiation. The asymmetric doping structure also produced intermediate energy levels that decreased the DCN‐FeCu work function under microwave irradiation, enabling DCN‐FeCu to cross the photoelectric effect barrier at a low‐energy microwave level (2.45 GHz), thus producing microwave catalytic properties. The layer number‐dependent microwave interlayer electron migration generated by this asymmetric doping modulation was demonstrated by modulating the layer number and verified by the asymmetric doping of FeCuMn trimetals. Thus, efficient catalytic behavior under microwave excitation can be achieved by the structural modulation of the asymmetric doping of 2D materials under the action of a microwave field.

## 2 Results and Discussions

### 2.1 Characterization of CN‐FeCu and DCN‐FeCu

**Figure 1:**
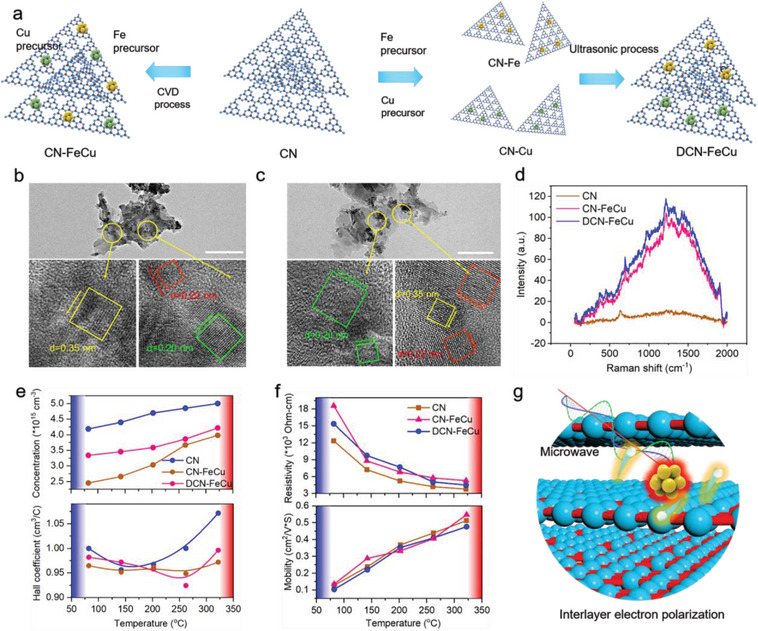
Structural characterization of DCN‐FeCu and measurement of carrier properties. a) The preparation diagram of CN‐FeCu and DCN‐FeCu. b,c) High‐resolution TEM images of CN‐FeCu and DCN‐FeCu. The scale bar = 100 nm. d) Raman spectra of CN, CN‐FeCu, and DCN‐FeCu. e) Resistivity and carrier concentration of CN, CN‐FeCu, and DCN‐FeCu. f) Carrier mobility and Hall coefficient of CN, CN‐FeCu, and DCN‐FeCu. g) The mechanical diagram of interlayer electron polarization under microwave irradiation.

**Figure** **1**a shows a schematic diagram of CN modified by normal Fe and Cu doping (CN‐FeCu) and asymmetric bimetallic doping. We prepared CN‐FeCu using a one‐step chemical vapor deposition (CVD) preparation method with CN, Fe, and Cu precursors. We then prepared CN‐Fe and CN‐Cu with Fe and Cu precursors, respectively, and obtained DCN‐FeCu through ultrasonication. Introducing asymmetric metal nanoclusters for doping‐induced interlayer electron polarization and the migration of interlayer electrons through microwave‐generated interlaminar charge transport, thus achieving efficient microwave catalysis. Transmission electron microscopy (TEM) images of CN‐FeCu and DCN‐FeCu revealed tiny particles, which represented the doping of heavy atoms (Fe and Cu) with larger atomic numbers (Figure 1b,c), but the TEM image of CN revealed no similar particles (Figure S1a, Supporting Information). High‐resolution transmission electron microscopy (HRTEM) images of CN‐FeCu and DCN‐FeCu showed the 002 crystal plane of CN (0.35 nm), a 110 crystal plane of the Fe nanoclusters (0.20 nm, bcc phase), and a 111 crystal plane of Cu nanoclusters (0.22 nm; Figure 1b,c). However, only the 211 crystal plane of CN was revealed (Figure S1b, Supporting Information). Moreover, the elemental mapping images of DCN‐FeCu showed a uniform distribution of C, N, Fe, and Cu elements, indicating the successful doping of the FeCu nanoclusters (Figure S1c, Supporting Information). In addition, the X‐ray diffraction (XRD) spectra after Fe and Cu doping showed that CN, CN‐FeCu, and DCN‐FeCu had only graphite‐like layer structure diffraction peaks (211 crystal plane; Figure S1d, Supporting Information). This proved that no new phase was generated by the doping of Fe and Cu, but the low content of Fe and Cu doping (w% of Fe/Cu:CN < 2%) produced no Fe and Cu crystalline surfaces that were observable from the diffraction peaks. Therefore, we proceeded to verify the presence of Fe and Cu nanoclusters using Raman spectroscopy (Figure 1d). Compared to CN, CN‐FeCu and DCN‐FeCu had higher scattering peaks, which resulted from the defects in CN caused by the doped FeCu nanoclusters. Next, we analyzed the valence state of the doped FeCu and bonding information using X‐ray photoelectron spectroscopy (XPS) spectroscopy (Figure S2, Supporting Information). After fitting, we found that the binding energies of Fe (708.1, 711.7, 716.2, 721.3, and 725.6 eV) corresponded to the binding energies of Fe^0^, Fe^2+^, and Fe^3+^ (Figure S2a, Supporting Information). The high‐resolution Cu XPS patterns of DCN‐FeCu showed that the binding energies of Cu after fitting were located at 928.6, 929.3, and 949.5 eV, corresponding to the binding energies of Cu^0^ and Cu^2+^ valence states (Figure S2b, Supporting Information). This also indicated that Fe and Cu were doped as nanoclusters in the triazine ring of the CN cell structure and bound to the N element in the CN. In addition, the binding energies were similar for the asymmetrically doped DCN‐FeCu and the conventionally doped CN‐FeCu, indicating that the forms of doped Fe and Cu were consistent (Figure S2c,d, Supporting Information).

### 2.2 Microwave‐Current Carrier Property of DCN‐FeCu

Considering that the valence of the doped Fe and Cu elements was higher than that of the original C atom, we observed residual unpaired electrons in Fe and Cu after coordination with N, which could easily be released to form free carriers under external excitation (heat, electricity, light, etc.).^[^ ^37^ ^]^ The average kinetic energy of the carriers at the interface and the average kinetic energy of the carriers at the thermal equilibrium were much higher than the average kinetic energy of the carriers, thus causing the excitation of carriers between and within the layers. First, we analyzed the carrier properties of CN‐FeCu and DCN‐FeCu without microwave irradiation (Figure 1e,f). The concentration increased correspondingly with increases in temperature, which we attributed to the multiple scattering of charge carriers due to the large number of lattice defects generated by the doping of Fe and Cu (Figure 1e). Compared with CN‐FeCu, DCN‐FeCu had a higher Hall coefficient (ability to respond to an electric field or microwave) and charge carrier concentration, but the difference in conductivity and charge carrier migration rate between CN‐FeCu and DCN‐FeCu was small (Figure 1e,f). This indicated that the overall electrical conductivity (i.e., the electron migration rate) was similar for the asymmetrically doped structure and the conventionally doped structure. However, the interlayer electron polarization effect caused by the structural differences between the two layers still had a huge impact on the carrier concentration, producing a difference in the Hall coefficiency—that is, a difference in the electron transport properties of the microwave field. We achieved this asymmetric doping of DCN‐FeCu by controlling the presence of only one type of nanocluster (Fe or Cu) on a layer of CN, thus generating inter and intralayer inconsistencies in CN that led to differences in polarization coupling between layers. The doping by introducing asymmetric metal nanoclusters induced interlayer electron polarization and the migration of interlayer electrons under microwave‐generated interlaminar charge transport, thus achieving efficient microwave catalysis (Figure 1g).

### 2.3 Microwave Electrochemical Properties

Since carrier migration was related to the microwave current and electrochemical impedance under microwave excitation, we verified this phenomenon using microwave electrochemical testing. First, according to the linear scan voltammetric (LSV) curve, the DCN‐FeCu had a larger microwave current value than the CN‐FeCu at the same voltage due to greater carrier migration under microwave irradiation (Figure S3a, Supporting Information). In contrast, CN exhibited the smallest microwave‐current value under the applied microwave irradiation, indicating that the doping of FeCu nanoclusters facilitated the generation of microwave‐excited electrons. Based on the electrochemical impedance spectrum (EIS), the impedance of DCN‐FeCu was lower than that of CN and CN‐FeCu (Figure S3b, Supporting Information), indicating the enhanced charge transfer rate of DCN‐FeCu and explaining the enhanced microwave electron excitation capability after doping. To further determine the semiconductor type of DCN‐FeCu after doping, we performed a Mott–Schottky test (Figure S3c,d, Supporting Information). The flat‐band potentials of CN‐FeCu and DCN‐FeCu were −0.33 and −0.16 V, respectively, indicating that FeCu‐doped CN was a p‐type semiconductor and showing that CN‐FeCu and DCN‐FeCu had an intrinsic carrier type with positively charged holes. Thus, the generation of electrons under microwave irradiation mainly originated from excitation jumps under microwave irradiation, rather than from the migration of intrinsic carriers.

### 2.4 Measurement of Microwave Absorption Properties of DCN‐FeCu

**Figure 2:**
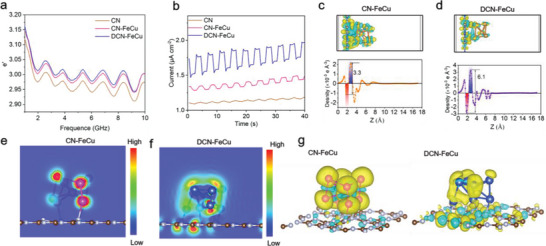
Nonlinear properties of electron delocalization domains due to interlayer polarization and microwave carrier transport. a) Real part of the dielectric constants of CN, CN‐FeCu, and DCN‐FeCu. b) Microwave current of CN, CN‐FeCu, and DCN‐FeCu generated under microwave irradiation (6 W, pulsed mode). c) Side view of the differential charge densities of CN‐FeCu and DCN‐FeCu and the corresponding charge density value curves. The equivalence surface is taken as 0.005 eV Å^−3^. Yellow meant the electron accumulation region and blue meant the electron dissipation region. d) Top view of the surface polarization of CN‐FeCu and DCN‐FeCu. e–g) Front view of the surface polarization of CN‐FeCu and DCN‐FeCu. Yellow meant the highly polarized region and blue meant the low polarized region.

The dielectric constant of DCN‐FeCu directly explains the microwave absorption properties of DCN‐FeCu. Under an alternating microwave field, the dielectric constant *ε* is generally expressed in a complex form (i.e., *ε* = *ε*′ − *jε*″).^[^ ^32^, ^38^ ^]^ The real part of the complex dielectric constant represents the ability to store microwave energy, whereas the imaginary part represents the ability to consume microwave energy.^[^ ^38^ ^]^ The magnitudes of the imaginary parts *ε*″ of the dielectric constants of CN, CN‐FeCu, and DCN‐FeCu were similar (Figure S4, Supporting Information, and **Figure** **2**a). However, the *ε*′ of DCN‐FeCu decreased relative to the original CN and was also smaller than that of CN‐FeCu, indicating that the asymmetrically doped DCN‐FeCu structure had better dielectric properties than the conventionally doped CN‐FeCu due to DCN‐FeCu having a stronger polarization speed and faster response rate under microwave irradiation, and thus, stronger dielectric loss.^[^ ^30^ ^]^ The sp^2^ conjugated structure of the C—N bond, the interlayer *π*–*π* conjugated structure of DCN‐FeCu, and the doped FeCu nanoclusters, together, formed a complete conductive network. Furthermore, we analyzed the ability of DCN‐FeCu to convert charge carriers following microwave absorption by measuring the microwave current generated by microwave excitation (Figure 2b). It was obvious that CN produced almost no microwave current under microwave irradiation, but both CN‐FeCu and DCN‐FeCu produced corresponding microwave currents, and DCN‐FeCu had a higher microwave current at 0.26 µA cm^−2^ than CN‐FeCu at 0.07 µA cm^−2^. This indicated that DCN‐FeCu had the greatest capability to produce microwave carriers under microwave conditions.

### 2.5 Interlayer Charge Transport Based on Electron Polarization

Due to the uncoordinated electron structure in the presence of Fe and Cu CN‐doped ions, electron polarization occurred under the electric field generated by microwave irradiation. Although the overall structure of DCN‐FeCu appeared electrically neutral, the bond lengths of Fe—N, C—N, Fe—Fe, Cu—N, C—N, and Cu—Cu were unequal. Following the excitation leap of electrons due to microwave irradiation, the asymmetric molecular structure of DCN‐FeCu and the unequal bond length changes made the electron distribution of the excitation leap noncoherent. Considering that the relaxation time of holes was extremely short compared to that of electrons, we analyzed the density distribution of electrons rather than holes.^[^ ^39^ ^]^ According to the differential charge densities of CN‐FeCu and DCN‐FeCu, it was obvious that the charge region (aggregate width of red and blue charge distribution) was 1.6 Å and remained unchanged (Figure 2c,d). However, the charge density of DCN‐FeCu (height difference of charge distribution in red plus blue) increased from 3.3 × 10^−3e^ to 6.1 × 10^−3e^ Å^−3^. The charge density in the CN region became more negative, and the charge density in the FeCu nanocluster region became more positive; thus, the direction of charge transfer corresponded with the FeCu nanocluster flowing to CN. This result illustrates that DCN‐FeCu (i.e., symmetric) doping favored the redistribution of electrons at the interface. The charge flow direction at the interface formed a charge depletion region (i.e., a built‐in electric field), the presence of which facilitated the excitation and migration of polarized electrons and favored interlayer charge transport (like FeCu‐CN–FeCu‐CN).

Regarding the corresponding mechanism, to further analyze the polarization characteristics of interlayer electrons, we investigated the spatial distribution of electron polarization (front view, Figure 2e,f; side view, Figure 2g). Compared to CN‐FeCu, DCN‐FeCu had a clear spatial distribution of polarization in the vertical direction in the occupied state. In addition, the polarization (the value of the difference between the highest and lowest chromaticity of the pseudo color) of the asymmetrically doped DCN‐FeC was stronger than that of CN‐FeCu. Interlayer coupling had a direct effect on the interlayer electron‐leaving domains and charge transport. In turn, varying the microwave field strength led to consistent dipole moment strength and microwave power, indicating that DCN‐FeCu's interlayer electron polarization rate was significantly microwave dependent.

### 2.6 Microwave Modulated Energy Band Structure

**Figure 3:**
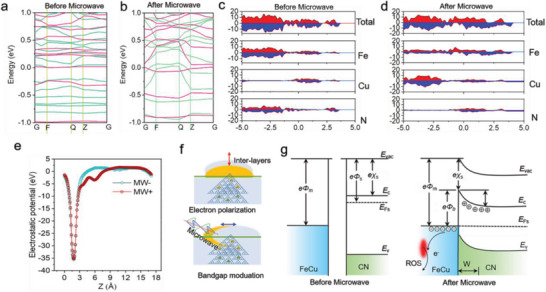
Microwave modulation of the band gap of DCN‐FeCu. a,b) Bandgap structure of DCN‐FeCu near the Fermi energy level before and after microwave treatment. c,d) Total and respective density of states of DCN‐FeCu before and after microwave treatment. e) The *W*_f_ of DCN‐FeCu before and after microwave treatment. f) The electron transport enhancement mechanism of DCN‐ FeCu structure. Interlayer charge transport in DCN‐FeCu is determined by a combination of the asymmetric structure and the modulation of the energy bands by the applied microwave. g) Schematic diagram of the energy bands of DCN‐FeCu doped metal and CN before and after contact. *E*_vac_ = vacuum energy, *E*_c_ = conduction band, *E*_v_ = valence band, *E*_fs_ = Fermi energy level, *F* = vacuum electrostatic potential, *c* = vacuum ionization energy, *V* = electric potential, *e* = electric charge, *W* = electron loss region.

Since the existence of a photoelectric potential barrier made it difficult for the microwave irradiation energy to release these electrons directly by leapfrogging, we speculated that the energy band structure of CN after FeCu doping contributed to the intermediate energy level, making it possible to achieve an energy band transition at low microwave energy between the band gaps of CN. As shown in **Figure** **3**a,b, more energy bands appeared near the top of the valence band of DCN‐FeCu after microwaving compared with DCN‐FeCu without microwave irradiation, mainly due to the repulsion between holes in the intermediate energy of FeCu confining the charge carriers to the top of the valence band, and more electrons crossing the Fermi energy level after microwave irradiation due to the departure of orbital electrons from the interface position. The doping of FeCu nanoclusters not only enhanced the absorption range of the spectrum but also reduced the energy band from 2.87 to 1.78 eV (Figure S5a,b, Supporting Information). The higher absorption value of DCN‐FeCu compared to CN‐FeCu was probably due to the multiple reflections of photons at the interface. To investigate the effect of the orbitals of the doped atoms on the energy band, we used the projected density of the DCN‐FeCu states for the analysis (Figure 3c,d). The impurity atom FeCu played a major role in the Fermi energy level for the electrons in the d orbitals; 4d, which is local in nature, has a cruising nature due to orbital hybridization and forms a continuous narrow energy band at the Fermi energy level. The reduction of the band gap was mainly due to the strong orbital hybridization of Fe 4d, Cu 4d, and N2, which resulted in impurity energy levels connecting with the valence band. Further analysis of the results showed that microwave irradiation was a feasible method for adjusting the band gap. Moreover, the heavily dominated energy level generated by microwave excitation narrowed the photoelectric potential barrier at the Fermi energy level, enabling it to be excited by a low‐energy microwave and causing electron leaps. The charge polarization on the surface of DCN‐FeCu under microwave irradiation‐induced changes in the surface dipole layer, thus changing the surface electron‐binding energy (i.e., changing the work function). Moreover, we investigated the effect of microwaves on the energy band structure of DCN‐FeCu by analyzing the work function (Figure 3e). The work function of DCN‐FeCu clearly decreased from 4.45 to 3.70 eV after microwaving, indicating that hybridized electrons of doped FeCu were injected into the CN conduction band, thus reducing the energy difference between the CN conduction band and the Fermi energy level of FeCu.

### 2.7 Enhancement Mechanism of Microwave Catalysis

Next, we analyzed the mechanism of microwave enhancement for the catalytic line of DCN‐FeCu using an energy band structure (Figure 3f). Before microwave application, relatively few CN electrons could cross the photoelectric potential barrier to reach the conduction band and transfer to the FeCu nanoclusters. After the formation of an interfacial heterojunction between CN and FeCu, band bending occurred at the interface due to the difference in the Fermi energy levels required to reach equilibrium. At the CN‐FeCu nanocluster interface, electrons were transferred from the FeCu side to the CN side, resulting in a negative charge on the CN side and a positive charge on the FeCu side. The charge gradually accumulated at the CN surface of the interface, resulting in an electrical FeCu field flowing to the CN. The thickness of the space charge region in CN was at the micron scale due to the limitation of the free charge density following contact between FeCu and CN; thus, the bending of the energy band under microwave irradiation occurred at the surface of CN rather than the surface of the FeCu nanoclusters. The reduction of the DCN‐FeCu work function by orbital hybridization with partial intermediate energy levels and more delocalization under microwave irradiation made it easier for the CN electrons to cross the photoelectric potential barrier, generating more microwave electrons and then transferring them to the FeCu nanoclusters. Therefore, the enhanced charge transfer capability of DCN‐FeCu was mainly due to the interlayer electron polarization caused by the asymmetric structure and modulation of the energy band by microwave irradiation (Figure 3g).

### 2.8 Layer and Dopant Metal‐Dependent Charge Transport

**Figure 4:**
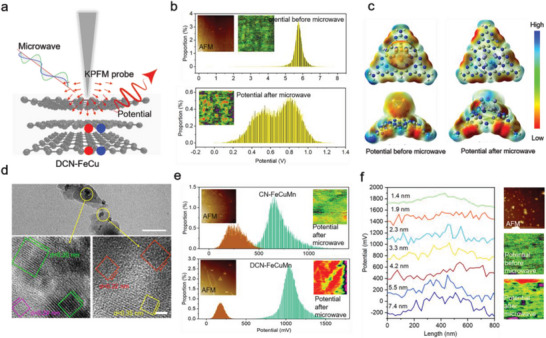
Measurement of layer number dependence and pervasiveness of trimetal by in situ KPFM technique. a) Schematic diagram of the potential change on the surface of DCN‐FeCu detected by in situ KPFM. b) The total surface potential distribution of DCN‐FeCu before and after microwave treatment. Insets are corresponding AFM and KPFM images. c) The surface electrostatic potential values of DCN‐FeCu before and after microwave application. d) High‐resolution TEM images of DCN‐FeCuMn. The scale bar = 100 nm. e) The total potential distributions of CN‐FeCuMn and DCN‐FeCuMn before and after microwave treatment, respectively. The inset showed the AFM and KPFM images. f) The potential variation curves of DCN‐FeCuMn at different thicknesses.

The microwave performance of asymmetrically doped DCN‐FeCu was mainly influenced by modulation of the energy band structure under microwave irradiation, which promoted interlayer charge transport, and the number of layers of 2D DCN‐FeCu also played a key role in charge transport. Under the action of the microwave, the energy state of the carriers generated in the lattice field was higher than that without the microwave. Therefore, the carriers along the interlayer caused a cumulative potential change in the lattice field (i.e., the interlayer potential difference of DCN‐FeCu). As shown in **Figure** **4**a, we detected the interlayer potential difference of DCN‐FeCu using an in situ Kelvin probe force microscope (KPFM). After microwave irradiation, the DCN‐FeCu interlayer potential was significantly enhanced, increasing from an average of 0.57 to 0.71 V (Figure 4b). The magnitude of the surface electrostatic potential (ESP) represents the magnitude of the polarization field and can directly affect the distribution of interlayer carriers and the migration rate. Thus, to determine why the number of layers affected electron transport, we examined the ESP of CN‐FeCu (Figure 4c). The surface ESP of the asymmetrically doped DCN‐FeCu increased from −0.05 to 0.08 mV compared to the conventionally doped CN‐FeCu, and the stronger electron polarization on the surface of the asymmetrically doped DCN‐FeCu led to enhanced interlayer coupling. On the one hand, the interlayer coupling changed the coordination interaction between CN and FeCu and enhanced the interatomic orbital hybridization, thus increasing the ESP of the surface. On the other hand, according to the band gap structure shown in Figure 3, the band gap of DCN‐FeCu was bent downward after being microwaved, and when the Fermi energy level crossed the center of the forbidden band, the surface positive charge density of DCN‐FeCu exceeded the surface negative charge density, at which time the depletion layer of the DCN‐FeCu interface was in a majority carrier antipattern state, generating a depletion layer with built‐in potential, offsetting some of the band bending, and leading to an increase in ESP.

### 2.9 The Asymmetrically Doping Universality of Trimetallic FeCuMn‐CN

To confirm the universality of asymmetric doping, we investigated the universality of the surface electrons of conventional FeCuMn trimetal‐doped CN (CN‐FeCuMn) and asymmetric FeCuMn‐doped CN (DCN‐FeCuMn). The HRTEM images of DCN‐FeCuMn revealed a 110‐crystal plane for the Fe nanocluster (0.20 nm), a 111‐crystal plane for the Cu nanocluster (0.22 nm), and a 112‐crystal plane for the Mn nanocluster (0.24 nm; Figure 4d). By comparing the surface potentials before and after microwaving, we found that CN‐FeCuMn and DCN‐FeCuMn increased 1.8‐ and 5.1‐fold, respectively, indicating that the interlayer potential of DCN‐FeCuMn increased greatly under microwave irradiation (Figure 4e). Thus, the trimetallic asymmetric doping of CN created greater interlayer electron polarization than conventional doping, which is favorable for electron generation and migration. Moreover, the potential also increased with increases in the number of layers (Figure 4f). The radical detection results showed that DCN‐FeCuMn produced 5.1‐fold more reactive oxygen species (ROS) than CN‐FeCuMn, with optimal catalytic activity (Figure S8a, Supporting Information). Based on the multiplication of ·O_2_^−^, it was clear that both CN‐FeCuMn and DCN‐FeCuMn produced ·O_2_^−^ radicals, which multiplied 1.7‐ and 4.5‐fold after microwaving (Figure S8b, Supporting Information). For the DCN‐FeCuMn structures with different layer numbers, the potential difference of interlayer electrons increased nonlinearly with increases in the number of layers, indicating that the asymmetric doping structures with multiple layer numbers favored 3D charge transport. The nonlocal degree of electrons decreased with increases in the number of layers, the extension of atomic orbitals became weaker, and the overall bonding also increased. Consequently, the orbital hybridization ability of CN and FeCuMn became stronger, which in turn affected the electron polarization between the layers and influenced the 3D charge transport between the layers.

### 2.10 Catalytic Pathway and Mechanism Analysis

**Figure 5:**
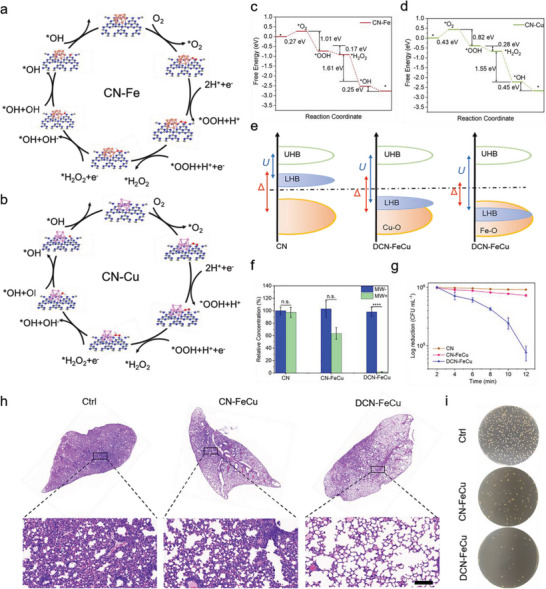
Microwave catalytic mechanisms of DCN‐FeCu and therapy toward bacterial infection pneumonia. a,b) The catalytic pathways located in the Fe active site and Cu active site. c,d) Gibbs free energy changes located in the Fe active site and Cu active site. e) Mechanistic analysis of the microwave‐catalyzed reaction of DCN‐FeCu. f) The degradation kinetics of CN‐FeCu and DCN‐FeCu toward MB. The significant difference in the different groups was compared with one‐way ANOVA. *p < 0.05, **p < 0.01, ***p < 0.001. *n* = 3. g) The antibacterial kinetics curves of CN, CN‐FeCu, and DCN‐FeCu for *Staphylococcus aureus* under microwave irradiation. h) The lung H&E images of *S. aureus* (10^7^ CFU mL^−1^) infected mice after PBS (Ctrl), CN‐FeCu, and DCN‐FeCu treatment at 2 days. The scale bar was 50 µm. i) The corresponding *S. aureus* counts in the infected lungs at 2 days of treatment.

We then further examined the types of ROS generated by microwave catalysis using oxidation analysis of different types of trapping agents. We used a DCFH probe to capture the total ROS, and nitroblue tetrazolium (NBT) to capture the ·O_2_^−^ (Figure S6a,b, Supporting Information). DCN‐FeCu generated 2.3‐fold more ROS than CN‐FeCu and achieved optimal catalytic activity (Figure S6a, Supporting Information). Based on the multiplication of ·O_2_^−^, it was clear that both CN‐FeCu and DCN‐FeCu produced ·O_2_^−^ radicals, and their multiplication after microwaving was 2.1 and 6.3 times, respectively, indicating that the main ROS produced by CN‐FeCu and DCN‐FeCu was ·O_2_^−^. Next, we used DFT to analyze the possible active site catalytic pathways of DCN‐FeCu under microwave irradiation (**Figure** **5**a,b). Because both Fe and Cu on the FeCu nanocluster can produce catalytic effects, we considered the catalytic reactions in the Fe and Cu nanoclusters separately and observed corresponding changes in the Gibbs free energy. O_2_ was first adsorbed on the CN‐Fe/Cu active site surface and formed *O_2_, and *O_2_ was then reduced to the transition state of *OOH by binding two protons, later forming *H_2_O_2_ after binding another proton. After dissociating one OH^−^, *H_2_O_2_ formed *OH and finally dissociated OH^−^ back to the starting state (Figure 5c,d). Analysis of the reaction pathways of these two active sites from a thermodynamic point of view showed that the reaction step with the highest energy barrier was the O_2_ adsorption process (highest potential barriers CN‐Fe at 0.27 eV and CN‐Cu at 0.43 eV); therefore, it was the probable catalytic pathway. Because the reaction potential barrier of CN‐Fe was the lowest, we presumed that the microwave‐catalyzed reaction of DCN‐FeCu occurred mainly on the surface of Fe.

The potential difference between (Fe/Cu‐O) and (Fe/Cu‐O)* depended mainly on the electronegativity difference between the doped metal ions and the oxygen atoms.^[^ ^40^ ^]^ For doped transition metals, further Mott–Hubbard splitting can occur due to electron repulsion inside the metal d orbitals, forming an electronically occupied low‐Hubbard‐band (LHB) and an empty‐state upper‐Hubbard‐band (UHB), respectively.^[^ ^39^ ^]^ The transition metal d–d Coulomb interaction was inversely proportional to the orbital volume and therefore depended greatly on the valence state of the metal. An increase in metal valence reduced the electronegativity difference and enhanced the d–d Coulomb interaction. Thus, in the catalytic process, as the doped metal valence state increased, the metal LHB dropped below the O 2p band, favoring ROS generation (Figure 5f). Therefore, the rate of microwave catalysis was mainly determined by the combined valence state of the doped metal and the doping structure.

We next analyzed the catalytic performance of CN‐FeCu and DCN‐FeCu by comparing changes in the bacterial colony numbers of *Staphylococcus aureus* before and after microwaving (Figure 5f). Increases in microwaving time led to DCN‐FeCu achieving optimal decomposition efficiency for methyl blue (MB) organic pollutants under microwave irradiation at 98.4%, which was much higher than the 38% decomposition efficiency of CN‐FeCu. Similarly, DCN‐FeCu showed significant bactericidal efficiency and outperformed CN‐FeCu and CN. The almost zero changes in the colony count for the CN group indicated that microwaving alone had no bactericidal effect on *S. aureus*. In contrast, the colony count for DCN‐FeCu decreased from 10^6^ CFU mL^−1^ to 7.2 × 10^4^ CFU mL^−1^ after 12 min of microwave irradiation, which was significantly lower than the colony count for the CN‐FeCu group (7.6 × 10^5^ CFU mL^−1^), indicating that DCN‐FeCu had optimal bactericidal efficiency under microwave irradiation.

We further evaluated the catalyst stability of DCN‐FeCu by measuring the XPS peaks before and after microwave irradiation (Figure S7a–c, Supporting Information). We found that the intensity of the characteristic peaks of Fe and Cu and the position of the binding energy did not change significantly before and after the microwaving of the samples, indicating better structural stability of DCN‐FeCu for microwave catalysis.

### 2.11 Rapid Therapy toward *S. aureus* Infected Pneumonia

To evaluate the antibacterial efficacy of CN‐FeCu and DCN‐FeCu in vivo, we built an *S. aureus‐*infected mouse pneumonia model. Based on the results of the 2 d treatment after *S. aureus* infection, the lung tissue in the (PBS‐treated) control group still showed considerable inflammation. Treatment with CN‐FeCu alleviated inflammation to some degree, but not as much as DCN‐FeCu due to the excellent antibacterial ability of microwaved DCN‐FeCu clearing most of the lung bacteria and thus attenuating the infection. At the end of the treatment, we then quantitatively analyzed the bacterial colonies within the lung tissue. The in vivo antibacterial efficiencies of CN‐FeCu and DCN‐FeCu were 73.3% and 98.2%, respectively.

## 3 Conclusions

In this study, we designed asymmetric metal‐nanocluster‐doped 2D g‐C_3_N_4_, which greatly enhanced microwave catalytic performance through interlayer electron polarization and microwave‐modulated energy band changes, leading to 3D charge transport. Interlayer interactions in 2D materials led to catalytic reactions, which depended on transport processes based on inter‐ rather than intralayer excitation. However, the modulation of charge transport between layers and the microwave catalysis of 2D materials by doping continues to be challenging. The asymmetric metal‐nanocluster doping enabled g‐C_3_N_4_ to generate interlayer carriers under microwave irradiation, leading to interlayer electron polarization and electron delocalization effects, thus enhancing the interlayer migration efficiency of electrons. The application of microwave irradiation allowed the asymmetric doping structure to generate intermediate energy levels through the hybridization of orbitals, leading to a decrease in work function that enabled DCN‐FeCu to cross the photoelectric effect barrier under low‐energy microwave irradiation (2.45 GHz), thereby providing microwave disinfection and pneumonia therapy. This asymmetric doping modulation produced layer‐dependent microwave electronic excitation, which was verified by the asymmetric doping of trimetallic FeCuMn. Therefore, efficient catalytic behavior under microwave excitation can be achieved by structurally modulating the ordered doping of 2D materials.

## 4 Experimental Section

### Material Synthesis

g‐C_3_N_4_ was prepared using a typical thermal decomposition method. Specifically, 5 g of urea was added to a porcelain boat and annealed in a muffle furnace at 550 °C for 2 h. The light yellow powder obtained after cooling was g‐C_3_N_4_. Then, 1 g of g‐C_3_N_4_, 0.1 g of FeCl_3_, and 0.05 g of NaBH_4_ were weighed and ground and placed the samples in a tube furnace and calcined in an N_2_ atmosphere at 600 °C for 4 h with the airflow rate maintained at 15 sccm. The product obtained was CN‐Fe. 1 g of g‐C_3_N_4_, 0.1 g of CuCl_2_, and 0.05 g of NaBH_4_ were weighed and ground and placed the samples in a tube furnace and calcined at 600 °C for 4 h in an N_2_ atmosphere with the airflow rate maintained at 15 sccm. The product obtained was CN‐Cu. 2 g of g‐C_3_N_4_, 0.1 g of CuCl_2_/FeCl_3_, and 0.05 g of NaBH_4_ were weighed and ground and placed the samples in a tube furnace and calcined at 600 °C for 4 h in an N_2_ atmosphere with the airflow rate maintained at 15 sccm. The product obtained was CN‐Cu/Fe or DCN‐FeCu.

### Characterization

A scanning electron microscope (S4800; Hitachi, Japan) and a transmission electron microscope (JEM‐2100F; JEOL, Japan) were used to characterize the surface and microscopic morphology of the samples. The physical and structural compositions of the samples were analyzed using X‐ray diffractometry and photoelectron spectroscopy. The light absorption capacity of the samples was analyzed using UV–vis‐NIR absorption spectroscopy, and the electrochemical properties were analyzed under microwave irradiation using an electrochemical workstation. Analysis of the wave electrocatalytic mechanism and carrier transport properties of the samples required theoretical calculations, and the wave absorption properties of the samples were analyzed using a microwave vector analyzer. A Hall effect tester was also used to analyze the carrier transport properties of the samples.

### Electrochemical Measurement

The electrochemical properties of the prepared samples were evaluated using a three‐electrode method with a CHI660E electrochemical workstation. The platinum electrode was used as the counter electrode, the Ag/AgCl electrode as the reference electrode, and configured 0.1 m saturated sodium sulfate solution as the electrolyte solution. First, the prepared samples were mixed with anhydrous ethanol and deionized water at a ratio of 1:4:5, and then prepared a 5 mg mL^−1^ solution, which was uniformly coated on the conductive glass and dried in an oven. The samples were irradiated with microwave power at 10 W and examined for microwave current, electrochemical impedance, and linear voltammetric curves.

### Antibacterial Assay

The sample NaCl solution was prepared, and co‐cultured with logarithmic phase bacterial solution (*S. aureus*). The sample bacterial solution was then irradiated at a microwave power of 6 W and recorded the temperature of the solution in real‐time. After 20 min of irradiation, the bacterial solution for plate coating was removed and calculated the antibacterial efficiency: antibacterial rate = (number of colonies in the control group – number of colonies in the experimental group/number of colonies in the control group.

### DFT Calculation

To determine the polarizability, theoretical calculations were performed at the density functional theory (DFT) level of the Perdew–Burke–Ernzerhof hybrid functional (PBE) method using a def2‐SVP basis set comprising Grimme's DFT‐D3(BJ), empirically dispersion corrected via the Gaussian 09 package. Harmonic vibrational frequencies were calculated at the same level to confirm that no imaginary frequencies existed in the molecules (i.e., they were located on the minima of the potential energy surface). Furthermore, the dipole moment and polarizability were determined based on the optimized molecules with PBE0‐D3(BJ)/def2‐TZVPP level of theory, extracting the values from Multiwfn 3.8. The molecular surface ESP was calculated using Gaussview, with an electron density value equal to 0.001 e/Bohr3, as suggested by Bader.

### Electromagnetics Test

A coaxial ring sample was prepared by mixing the sample with paraffin wax at a ratio of 2:3, and then scanned it using a microwave vector network analyzer (PNA‐N5244A; Agilent Technologies, USA) in the frequency range of 2–18 GHz.

### Animal Test

6‐week‐old mice were purchased from Tianjin Yi Sheng Yuan Biotechnology Co., Ltd. (approval number: SYXK2021‐0003), and conducted all experimental procedures in accordance with the standards of the Animal Ethics Association. The mice were divided into three groups: a PBS‐treated (control) group, a CN‐FeCu group, and a DCN‐FeCu group. The mice were then starved for 12 h prior to surgery and treated for a lung infection. The concentrations and amounts of *S. aureus* used to induce the infection were 10^7^ CFU mL^−1^ and 50 µL, respectively. Subsequently, the PBS‐treated group, CN‐FeCu group, and DCN‐FeCu group were subjected to nasal drip and microwave irradiation treatment for 10 min, observing the real‐time temperature using a thermal imager. Following 2 days of treatment, the mice were executed and analyzed the lung tissue pathology.

### Statistical Analysis

All the quantitative data in this work were analyzed using a one‐way analysis of variance (ANOVA). The *p*‐value less than 0.05 was considered statistically significant: *p < 0.05, **p < 0.01, ***p < 0.001, ****p < 0.0001. The numbers of all samples were kept at three, and the data were presented as means with standard deviations (SD). Besides, the figures were drawn with Origin software.

## Conflict of Interest

The authors declare no conflict of interest.

## Supporting information

Supporting Information

## Data Availability

The data that support the findings of this study are available from the corresponding author upon reasonable request.
