# Supplementary material for: Interlayer Electrons Polarization of Asymmetric Metal Nanoclusters/g‐C3N4 for Enhanced Microwave Therapy of Pneumonia
Source: Adv Sci (Weinh). 2023 May 10;10(21):2301817. doi: 10.1002/advs.202301817 (PMC10375082; doi:10.1002/advs.202301817)
Supplement: Supplementary file 1 — Supporting Information [file ADVS-10-2301817-s001.pdf]

ADVANCED SCIENCE
Open Access

Supporting Information

for *Adv. Sci.*, DOI 10.1002/advs.202301817

Interlayer Electrons Polarization of Asymmetric Metal Nanoclusters/g-C_3N_4 for Enhanced Microwave Therapy of Pneumonia

Yuan Li, Shuilin Wu, Yufeng Zheng, Zhaoyang Li, Zhenduo Cui, Hui Jiang, Shengli Zhu and Xiangmei Liu**

Supporting Information

Interlayer Electrons Polarization of Asymmetric Metal Nanoclusters/g-C_3N_4 for Enhanced Microwave Therapy of Pneumonia

Yuan Li, Shuilin Wu, Yufeng Zheng, Zhaoyang Li, Zhenduo Cui, Hui Jiang, Shengli Zhu, Xiangmei Liu**

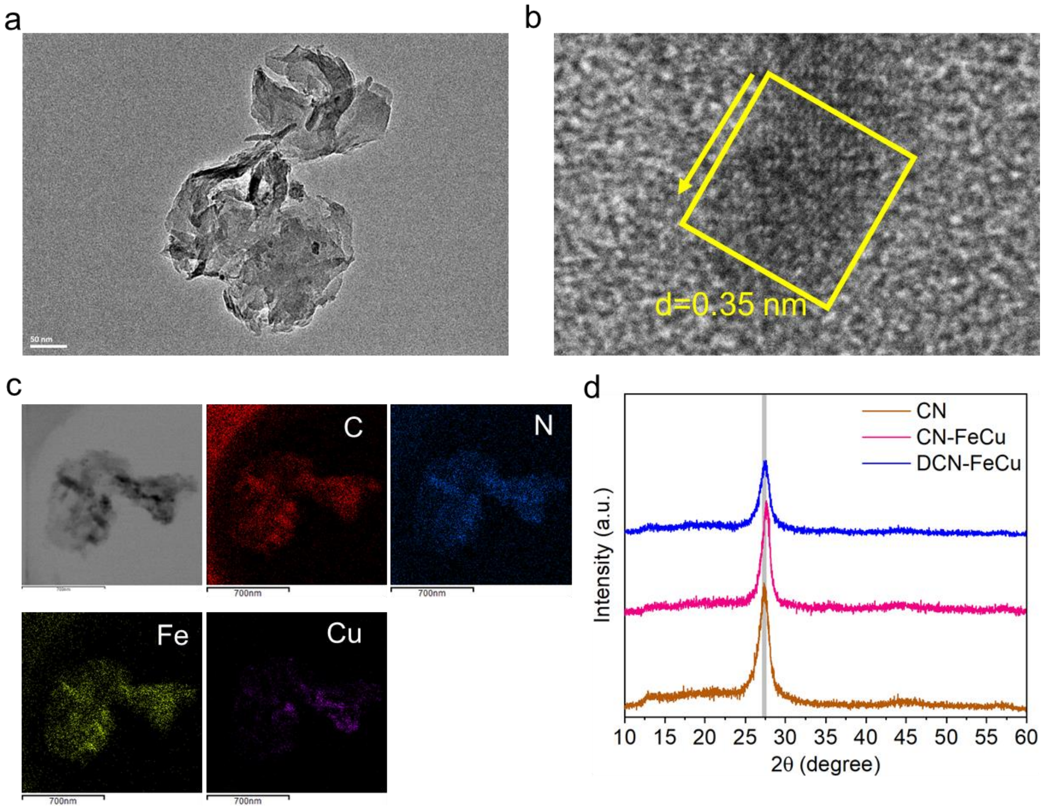

FigureS1. a-b, TEM and HRTEM images of CN. c, elemental mapping images of DCN-FeCu. d, the XRD spectra of CN, CN-FeCu, and DCN-FeCu.

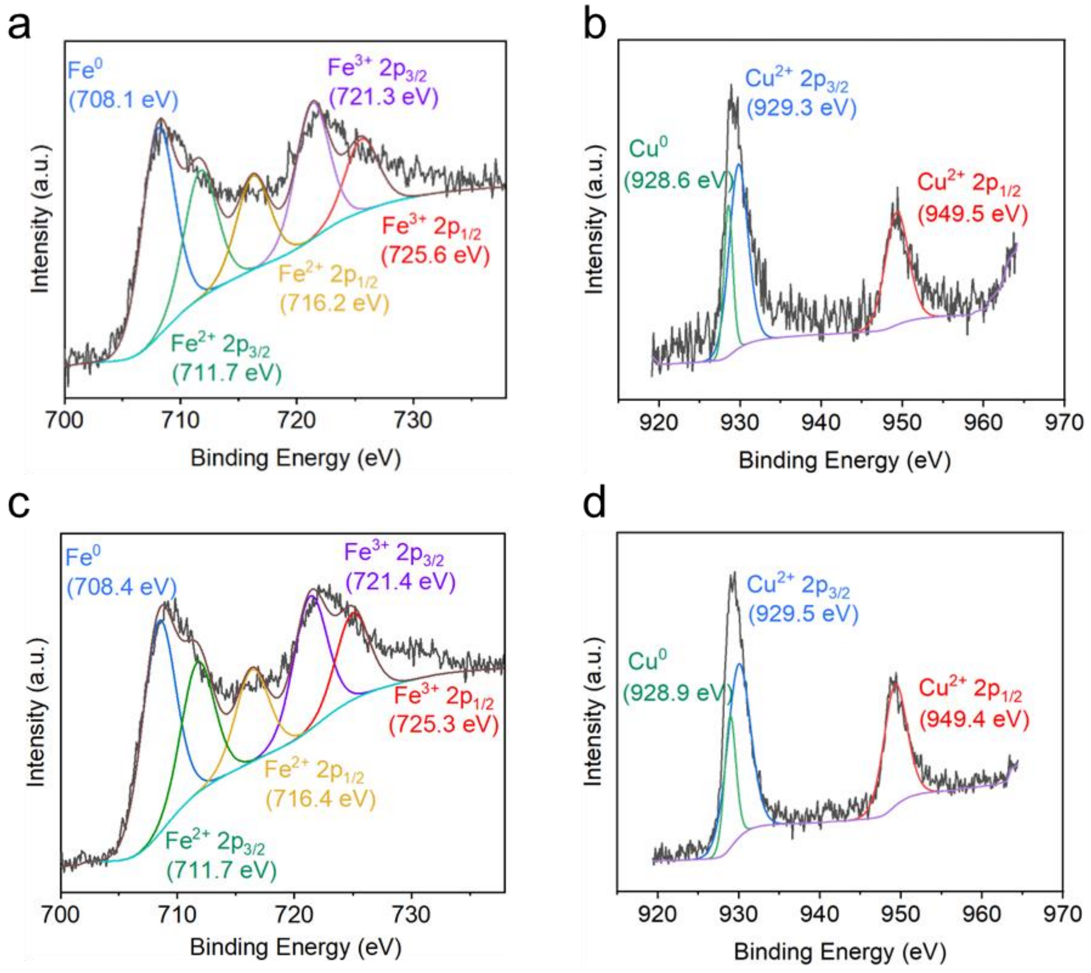

Figure S2. a-b, high-resolution XPS patterns of Fe and Cu elements for DCN-FeCu. c-d, high-resolution XPS patterns of Fe and Cu elements for CN-FeCu.

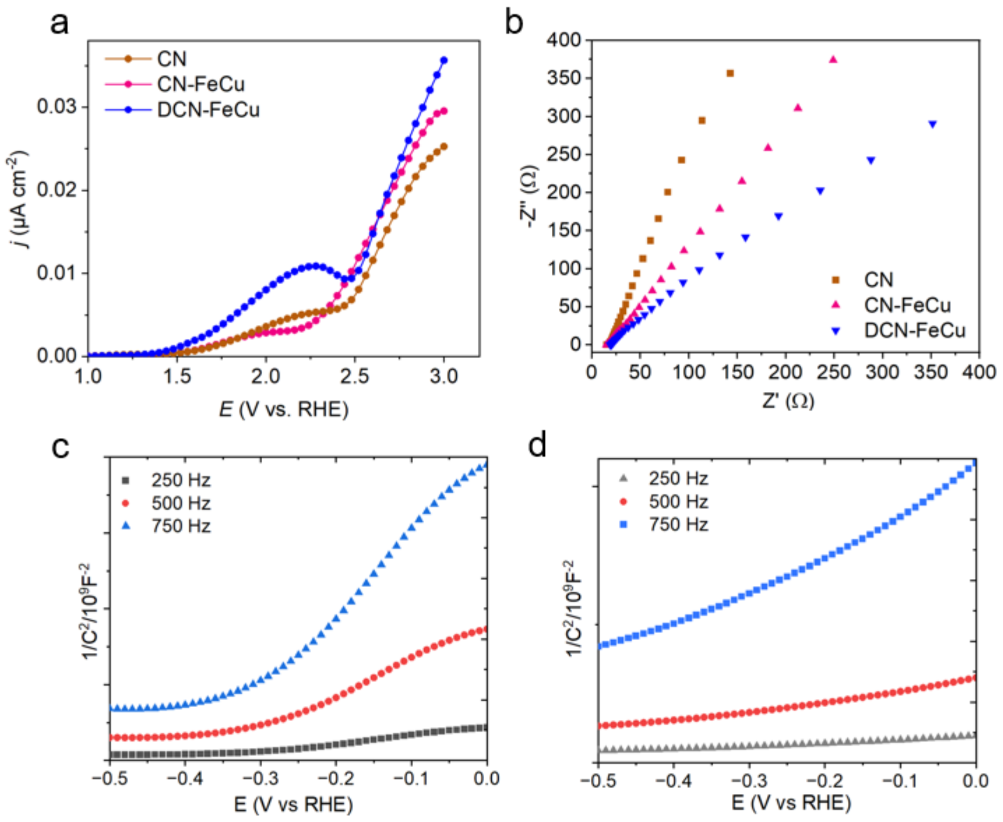

Figure S3. a, linear voltammetric scan curves of CN, CN-FeCu and DCN-FeCu. b, EIS curves of CN, CN-FeCu and DCN-FeCu. c-d, Mott Schottky curves of DCN-FeCu and CN-FeCu.

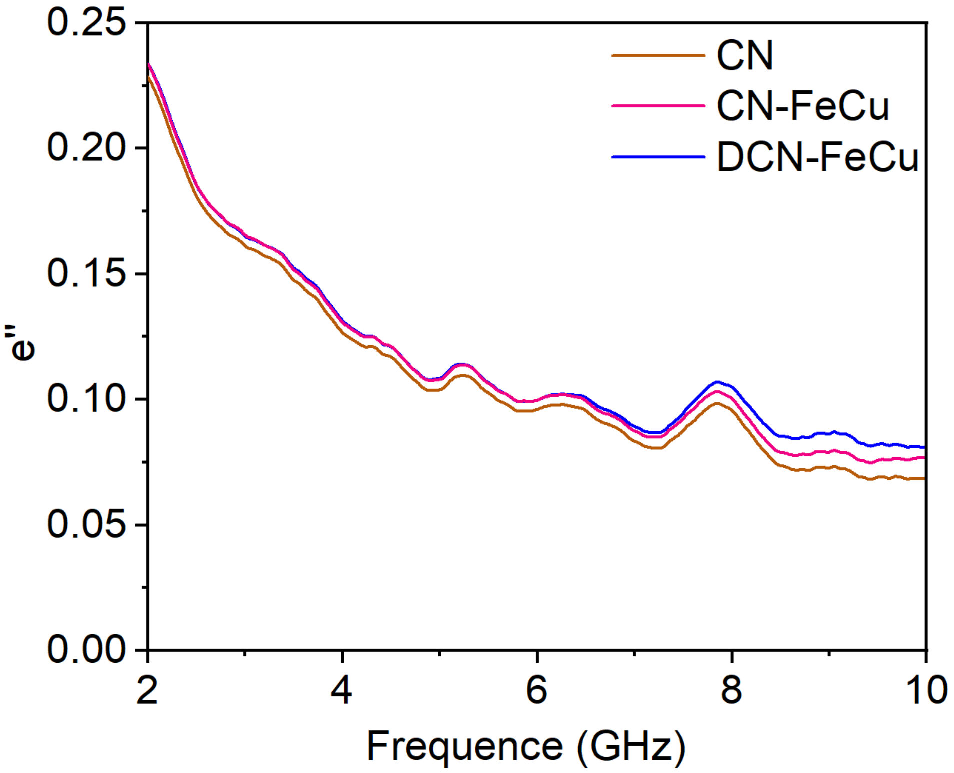

Figure S4. The imaginary part of the dielectric constants of CN, CN-FeCu and DCN-FeCu.

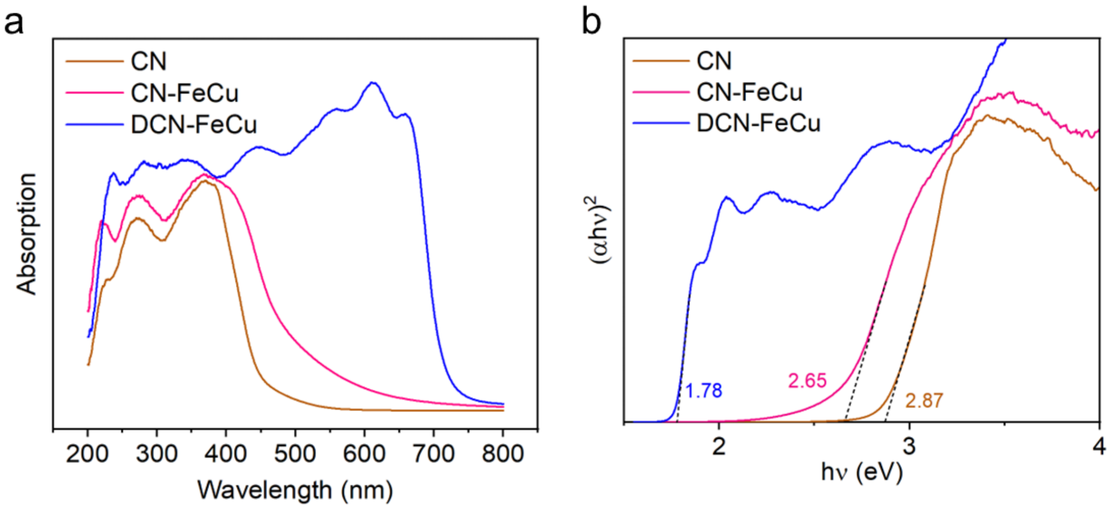

Figure S5. a, DRS spectra of CN, CN-FeCu and DCN-FeCu. b, band gaps of CN, CN-FeCu and DCN-FeCu calculated from the DRS spectra.

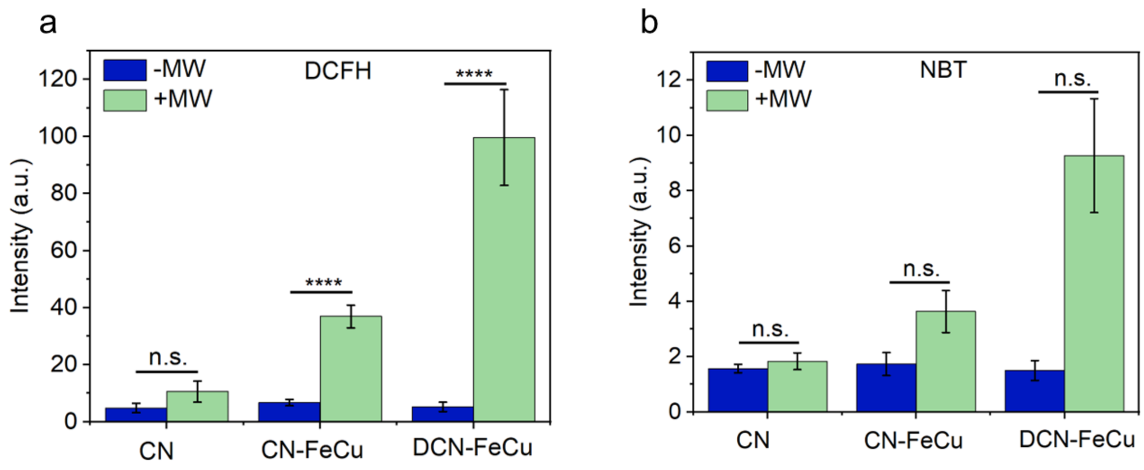

Figure S6. a, total ROS content of CN, CN-FeCu and DCN-FeCu under microwave irradiation. b, O_2^- content of CN, CN-FeCu and DCN-FeCu under microwave irradiation.

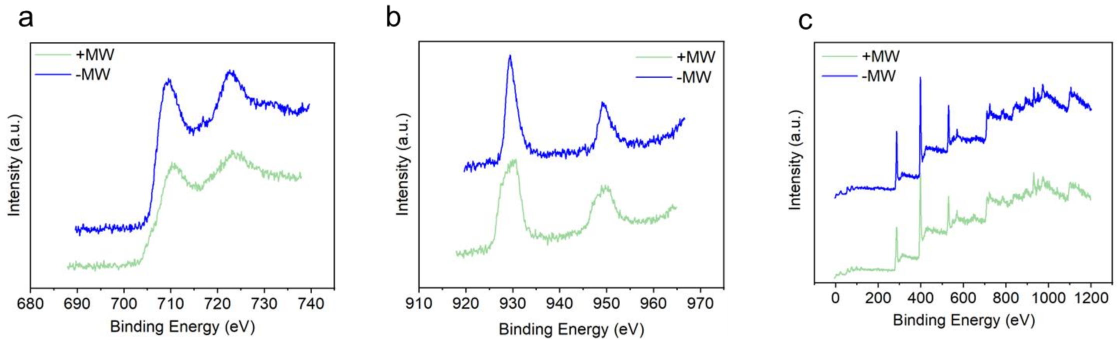

Figure S7. a, high-resolution XPS patterns of Fe before and after microwave for CN, CN-FeCu and DCN-FeCu. b, high-resolution XPS patterns of Cu before and after microwave for CN, CN-FeCu and DCN-FeCu. c, total XPS patterns of CN, CN-FeCu and DCN-FeCu before and after microwave treatment for CN, CN-FeCu and DCN-FeCu.

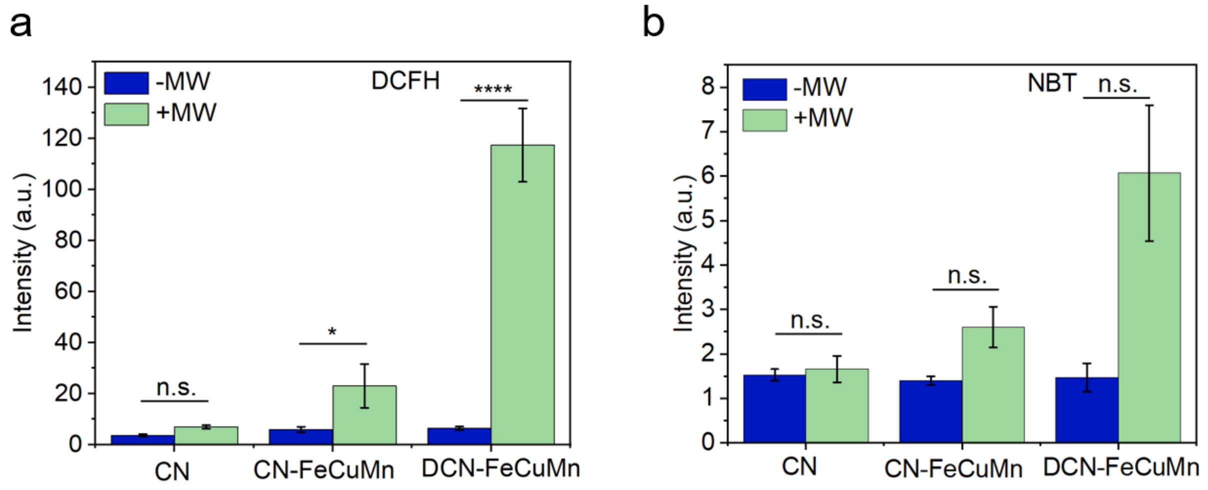

Figure S8. a, total ROS content of CN, CN-FeCuMn and DCN-FeCuMn under microwave irradiation. b, O_2^- content of CN, CN-FeCuMn and DCN-FeCuMn under microwave irradiation.
